# Human Articular Cartilage Progenitor Cells Are Responsive to Mechanical Stimulation and Adenoviral-Mediated Overexpression of Bone-Morphogenetic Protein 2

**DOI:** 10.1371/journal.pone.0136229

**Published:** 2015-08-20

**Authors:** Alexander J. Neumann, Oliver F. W. Gardner, Rebecca Williams, Mauro Alini, Charles W. Archer, Martin J. Stoddart

**Affiliations:** 1 AO Research Institute Davos, AO Foundation, Clavadelerstrasse 8, 7270, Davos Platz, Switzerland; 2 Cardiff School of Biosciences, Cardiff University, CF10 3 AX, Cardiff, Wales, United Kingdom; 3 Swansea School of Medicine, Swansea University, SA2 8PP, Swansea, Wales, United Kingdom; University of Zaragoza, SPAIN

## Abstract

Articular cartilage progenitor cells (ACPCs) represent a new and potentially powerful alternative cell source to commonly used cell sources for cartilage repair, such as chondrocytes and bone-marrow derived mesenchymal stem cells (MSCs). This is particularly due to the apparent resistance of ACPCs to hypertrophy. The current study opted to investigate whether human ACPCs (hACPCs) are responsive towards mechanical stimulation and/or adenoviral-mediated overexpression of bone morphogenetic protein 2 (BMP-2). hACPCs were cultured in fibrin-polyurethane composite scaffolds. Cells were cultured in a defined chondro-permissive medium, lacking exogenous growth factors. Constructs were cultured, for 7 or 28 days, under free-swelling conditions or with the application of complex mechanical stimulation, using a custom built bioreactor that is able to generate joint-like movements. Outcome parameters were quantification of BMP-2 and transforming growth factor beta 1 (TGF-β1) concentration within the cell culture medium, biochemical and gene expression analyses, histology and immunohistochemistry. The application of mechanical stimulation alone resulted in the initiation of chondrogenesis, demonstrating the cells are mechanoresponsive. This was evidenced by increased GAG production, lack of expression of hypertrophic markers and a promising gene expression profile (significant up-regulation of cartilaginous marker genes, specifically collagen type II, accompanied by no increase in the hypertrophic marker collagen type X or the osteogenic marker alkaline phosphatase). To further investigate the resistance of ACPCs to hypertrophy, overexpression of a factor associated with hypertrophic differentiation, BMP-2, was investigated. A novel, three-dimensional, transduction protocol was used to transduce cells with an adenovirus coding for BMP-2. Over-expression of BMP-2, independent of load, led to an increase in markers associated with hypertropy. Taken together ACPCs represent a potential alterative cell source for cartilage tissue engineering applications.

## Introduction

Hyaline articular cartilage possesses a limited intrinsic repair capacity. Cartilage defects that are not, or not sufficiently, treated render the cartilaginous tissue prone to further degeneration. Ultimately, this might result in the development of secondary osteoarthritis [1]. Currently, available treatment options, such as microfracture [2], mosaicplasty [3], soft-tissue grafts [4] or autologous chondrocyte transplantation [5] still fail to demonstrate reproducible success in articular cartilage regeneration.

Tissue engineering (TE) represents a promising alternative treatment option for articular cartilage defects [6]. The two most commonly used chondrogenic stimuli are bioactive factors and mechanical stimulation (reviewed for example in: [7–13]). However, in TE applications, the starting cell source is still a topic of debate [14].

Articular cartilage progenitor cells (ACPCs) represent a potential alternative cell source to bone-marrow derived mesenchymal stem cells (MSCs) and chondrocytes, and have been shown to be resistant to hypertrophy. They were first isolated, through differential adhesion to fibronectin, from the superficial zone of bovine articular cartilage [15]. In later work of the same group, these cells were further characterised [16, 17]. More recently it was demonstrated that these cells can be isolated from healthy and osteoarthritic human articular cartilage [18].

In 2011, McCarthy et al., performed the first direct comparison between MSCs and ACPCs using horse derived cells [19]. This study used a pellet culture model, which is currently considered the "gold standard" for *in vitro* chondrogenesis. In summary, it was shown that both cell types were able to undergo chondrogenesis, in the presence of low serum concentrations. However, MSCs expressed markers associated with hypertrophy, a problem commonly associated with MSCs, whereas ACPCs appeared to undergo a more stable chondrogenesis. Resistance to hypertrophy has also been demonstrated using human derived ACPCs [18].

Based on these encouraging results, we aimed to further investigate the chondrogenic potential of human ACPCs (hACPCs). The present study aimed to investigate whether hACPCs are able to respond to mechanical stimulation. In addition we aimed to investigate whether hACPCs maintained resistance to hypertrophy in the presence of bone morphogenetic protein 2 (BMP-2 overexpression).

A composite scaffold system, comprising of a fibrin hydrogel to maintain a 3D cellular environment and a polyurethane (PU) macroporous scaffold to provide bulk mechanical properties was used. While the fibrin/PU scaffold system is not optimal for TE purposes due to a significant release of matrix products into the medium, we have found it to be ideally suited to investigate chondrogenesis induced by complex multiaxial load due to its highly resilient behaviour and its ability to withstand interfacial shear. Complex mechanical stimulation was administered using a novel bioreactor system designed around tribological considerations [20]. It was demonstrated that the fibrin-PU composite system is comparable to the "gold standard" pellet culture model and that it compares favourably regarding expression of genes related to endochondral ossification when using human MSCs (hMSCs) [21]. It was further shown in this system that mechanical stimulation enhances endogenous production of both transforming growth factor beta- (TGF-β) 1 and TGF-β3 [22]. The production of TGF-β isoforms then leads to the chondrogenic induction of human MSCs, in medium lacking exogenous TGF-β1. More recently, it was demonstrated that shear forces play an important role in chondrogenesis of human MSCs [23].

A beneficial effect of retroviral-mediated over-expression of (BMP-2) and mechanical stimulation on the chondrogenesis of de-differentiated bovine chondrocytes has previously been demonstrated [24]. Recently, it was also shown that the combination of mechanical stimulation and adenoviral-mediated over-expression of BMP-2 harbours potential for *in vitro* chondrogenesis of hMSCs [25]. However, the use of bone marrow derived MSCs this chondrogenesis is more associated with a hypertrophic / endochondral phenotype [25, 26]

It was hypothesised that mechanical stimulation will lead, as already demonstrated for hMSCs [22], to an enhanced endogenous production of TGF-β1 and, thereby, induce chondrogenesis. Furthermore, we aimed to establish whether ACPCs were able to resist hypertrophy induced by overexpression of BMP-2.

## Materials and Methods

### Culture media

#### Growth medium

Dulbecco's modified eagle medium (DMEM) GlutaMax (Gibco, Carlsbad, CA) supplemented with 1mM sodium pyruvate, 0.1mM L-ascorbic acid 2-phosphate sesquimagnesium salt hydrate (AA), 10mM HEPES buffer, 0.5mg/ml additional glucose, 10% foetal bovine serum (Gibco, Carlsbad, CA), 1% penicillin/streptomycin (P/S), 5ng/ml basic fibroblast growth factor and 1ng/ml TGF-β2 (both Peprotech, Rocky Hill, NY).

#### Serum-free DMEM

Serum-free, high glucose (4.5 g/L) DMEM (Gibco, Carlsbad, CA) containing 1mM sodium pyruvate, 3.7g/L NaHCO_3_ and 1% P/S.

#### Chondropermessive medium

Serum-free DMEM supplemented with 1% MEM non-essential amino acids (Millipore, Billerica, MA), 1% insulin-transferrin selenium premix (Cyagen, Guangzhou, China), 50μg/ml AA, 10^-7^M dexamethasone. 5μM of 6-aminocaproic acid was added in order to prevent fibrin degradation [27]. As the bioreactor represents an open system, P/S was replaced with 100 μg/ml of Primocin (Invivogen, San Diego, CA).

### Biodegradable polyurethane scaffold preparation

Biodegradable, cylindrical (8x4 mm) PU scaffolds (pore size 90–300 μm) were synthesized as previously described [28]. Briefly, hexamethylene diisocyanate, poly(epsilon-caprolactone) diol and 1,4:3,6-dianhydro-D-sorbitol were reacted in a one-step solution polycondensation reaction. The scaffolds were prepared using the salt-leaching phase inverse technique with sodium phosphate heptahydrate dibasic salt as porogen and cut using the water jet technique.

### Articular cartilage progenitor cell isolation

hACPCs were isolated from full-depth human cartilage samples from the tibial plateaux of healthy donors obtained with relevant ethical approval and informed written patient consent (NHS Blood and Tissue bank, Liverpool, UK), as described elsewhere [18]. Briefly, chondrocytes were isolated by sequential digest with pronase (1 hour at 37°C, 70units/ml) and collagenase (3 hours at 37°C, 300units/ml). hACPCs were isolated from the chondrocyte population through differential adhesion to fibronectin.

### Articular cartilage progenitor cell culture

Initial DMSO frozen stocks of hACPSs were stored in Liquid nitrogen after 26.23 ± 1.43 population doublings (PDs). The cells were then further expanded in growth medium, with partial (50% of the total medium volume) medium changes 3 times per week, until 70–80% confluent. Seeding density was always 13 333 cells/cm^2^. The standard culture conditions were 37°C, 5% CO_2_ and 90% humidity in a CO_2_ incubator. During the second expansion phase, hACPCs underwent an additional 7.56 ± 0.36 PD.

### Propagation of a recombinant adenoviral vector coding for BMP-2

A first-generation, E1-, E3-deleted, serotype 5 adenoviral vectors carrying the cDNA for human BMP-2 (Ad.BMP-2; Vector Biolabs, Philadelphia, PA) was amplified in AD293 cells (Stratagene, Santa Clara, CA), purified over successive caesium chloride gradients and dialysed. The standard plaque assay on AD293 cells was used to quantify the viral titre.

### Articular cartilage progenitor cell seeding and Ad.BMP-2 transduction in 3D

Sub-confluent hACPCs (a total of 32.81 ± 2.33 PD after isolation) were harvested using trypsin, embedded into a fibrin hydrogel (17mg/ml fibrinogen and 0.5units/ml thrombin) and seeded (5 x 10^6^ cells per scaffold) into sterile PU scaffolds as previously described [21]. BMP-2 groups were transduced with Ad.BMP-2, applying a novel three-dimensional transduction protocol (BMP-2), as previously described [29]. A multiplicity of infection (MOI) of 5 was used. One MOI was defined as one infectious viral particle per cell. Un-transduced hACPCs served as controls as it has been previously shown that adenoviral control vectors do not induce a chondrogenic differentiation [30, 31]. The samples were pre-cultured for 3 days in 5 ml of chondropermessive medium.

### Scaffold culture and mechanical stimulation

The samples were cultured in 2.5 ml of chondropermessive medium with three medium changes per week. The samples were either kept under free-swelling conditions (unloaded) or with the application of mechanical stimulation (loaded). Mechanical stimulation was conducted on six days per week for 1 hour as described elsewhere [25]. A custom built bioreactor system was used [20]. Briefly, unconfined, dynamic compression (1Hz, 0.4–0.8 mm) and shear stress (1 Hz, ± 25°) were superimposed on a static offset strain of 0.4 mm, this is the equivalent of cycling between 5%- 10% strain. The experiment was independently repeated with cells from four different donors (♂ age 75, ♂ age 56, ♂ age 33 and ♀ age 30) with triplicates for each group (Table 1).

**Table 1:** Overview of the eight different experimental groups, which were tested in the study.

Group	Viral Transduction	Load	Time in three dimensional culture
1	No	No	7
2	No	Yes	7
3	No	No	28
4	No	Yes	28
5	First generation, E1-, E5-deleted, serotype 5 adenoviral vector carrying the cDNA for human BMP-2 (Ad.BMP-2)	No	7
6	Ad.BMP-2	Yes	7
7	Ad.BMP-2	No	28
8	Ad.BMP-2	Yes	28

### Sample collection

The medium of the three day pre-culture period was individually collected. Further, the culture medium was collected after medium changes and pooled for day 1–7, day 8–14, day 15–21 and day 22–28. After 7 or 28 days, scaffolds were vertically cut into two halves. One half was placed into 1 ml of TRI reagent and 5 μl of Polyacrylcarrier and stored at -80°C for subsequent RNA isolation.

The other half scaffold was digested with 1 ml of 0.5 mg/ml Proteinase K for 16 hours at 56°C. Afterwards, Proteinase K was heat-deactivated for 10 minutes at 96°C and the samples were stored at -20°C for subsequent biochemical analyses.

### BMP-2 and TGF- β1 ELISA

The concentration of BMP-2 and TGF-β1 were quantified using a quantitative Duo Set ELISA kit for human BMP-2 or human TGF-β1 (both R&D Systems, Minneapolis, MN) according to the manufacturer's protocol. Prior to the assay, latent TGF-β1 was activated according to the manufacturer's protocol.

### Gene expression analyses

Isopropanol and a high salt precipitation solution were used to precipitate the RNA. Reverse transcription was carried out using TaqMan reagents, 1 μg of RNA and a reaction volume of 20 μl. Real-time polymerase chain reaction (PCR) was performed using the 7500 real time PCR system (Applied Biosystems, Carlsbad, CA). Gene expression analyses were conducted using the comparative ΔΔC_t_ method and 18s RNA as internal control. The genes 18s RNA, sex-determining region Y-box 9 (SOX9), Notch 1, alkaline phosphatase (ALP) and parathyroid hormone-related protein (PTHrP) were detected using the commercial pre-developed TaqMan assay reagents (Applied Biosystems, Carlsbad, CA).

The oligonucleotide primers and probes for the remaining genes have previously been designed and validated using the Primer Express Oligo Design software version 1.5 (Table 2).

**Table 2:** Self-designed forward primers, reverse primers and probes used for real-time PCR. The primers and probes were designed and validated within our group using the primer express design software V.1.5 and were synthesized by Microsynth.

Gene	Primer forward	Primer reverse	Probe (5´FAM/3´TAMRA)
human collagenIA1	5'-CCC TGG AAA GAA TGG AGA TGA T-3'	5'-ACT GAA ACC TCT GTG TCC CTT CA-3'	5'-CGG GCA ATC CTC GAG CAC CCT-3'
human collagenIIA1	5'-GGC AAT AGC AGG TTC ACG TAC A-3'	5'-GAT AAC AGT CTT GCC CCA CTT ACC-3'	5'-CCT GAA GGA TGG CTG CAC GAA ACA TAC-3'
human collagenXA1	5'-ACG CTG AAC GAT ACC AAA TG-3'	5'-TGC TAT ACC TTT ACT CTT TAT GGT GTA-3'	5'-ACT ACC CAA CAC CAA GAC ACA GTT CTT CAT TCC-3'
human aggrecan	5'-AGT CCT CAA GCC TCC TGT ACT CA-3'	5'-CGG GAA GTG GCG GTA ACA-3	5'-CCG GAA TGG AAA CGT GAA TCA GAA TCA ACT-3'
human runt-related transcription factor 2	5'-AGC AAG GTT CAA CGA TCT GAG AT-3'	5'-TTT GTG AAG ACG GTT ATG GTC AA-3'	5'-TGA AAC TCT TGC CTC GTC CAC TCC G-3'
human bone-morphogenetic protein 2	5'-AAC ACT GTG CGC AGC TTC C-3'	5'-CTC CGG GTT GTT TTC CCA C-3'	5'-CCA TGA AGA ATC TTT GGA AGA ACT ACC AGA AAC TG-3'

### Biochemical analyses

The dimethylmethylene blue (DMMB) dye binding assay was used to quantify the amount of sulphated glycosaminoglycan (GAG) [32]. Chondroitin-4-sulfate from bovine trachea served as standard.

The Hoechst 33258 dye assay was used to quantify the total amount of DNA [33]. Calf thymus DNA was used as standard.

### Histology and immunohistochemistry

The scaffolds were fixed in 70% methanol at 4°C. Before cryosectioning, they were incubated for 12 hours at 4°C in 5% D(+)sucrose solution in phosphate buffered saline (PBS) and, afterwards, embedded in O.C.T compound (R.Jung GmbH, Nussloch, Germany). Cryosections (12 μm) were cut using the HM560 cryotome (Thermo Fischer Scientific, Waltham, MA). In order to visualise negatively charged proteoglycans, sections were stained with Safranin-O. Fast Green was used to counter-stain proteins. Weigert´s iron hematoxyline was used to stain the cell nuclei.

Additionally, the deposition of the matrix proteins collagen type II (Col II), collagen type X (Col X) and aggrecan was visualised by immunohistochemistry. Before immunolabelling for the aggrecan protein could be conducted, reduction and alkylation steps were necessary to expose a neo-epitope. The endogenous peroxidase activity was blocked with 0.3% peroxidase in 100% methanol and the sections were enzymatically pre-treated (0.025 U/ml of chondroitinase AC for aggrecan or 0.05 U/ml of hyaluronidase for Col II and Col X; both Sigma, St.Louis, MO). Next, sections were blocked with 5% normal horse serum and, subsequently, incubated with the primary antibodies (all 30 minutes at room temperature) against aggrecan (1-C-6, 8.167 μg/ml), Col II (CIICI, 1.67 μg/ml) (both Developmental Studies Hybridoma bank, university of Iowa, Iowa City, IA) and Col X (Col-10, 0.5 μg/ml, Sigma, St.Louis, MO). Negative control sections were treated with PBS + 0.1% Tween 20 instead. The Vectastain elite ABC kit mouse IgG and the ImmPACT DAB peroxidase substrate were used as detection system (both Vector Laboratories, Burlingame, CA). The cell nuclei were stained with hematoxyline. Finally, the slides were dehydrated in increasing concentrations of ethanol, cleared in xylene, embedded in Eukitt quick-hardening mounting medium (Fluka, St.Louis, MO) and cover-slipped.

### Image Acquisition

Image acquisition was conducted with the Axioplan 2 microscope, the AxioCamHR camera and the Carl Zeiss AxioCamHR V.5.07.03 software (all Carl Zeiss AG, Oberkochen, Germany).

### Statistical analyses

Statistical analyses were conducted with the SPSS software (SPSS 19, IBM, NY). The independent samples Kruskal-Wallis test was used to determine normality of each group. Equal variances between groups were tested using the Levene's Test of equality of error variances. A general linear model analysis of variance with a Games Howell Post-hoc analysis for unequal variances was used to detect significance of differences between the groups. To specifically determine the effect of load, a t-test was used to compare to unloaded control. Transformation by natural logarithm was applied for the gene expression analysis data. Descriptive results are displayed as mean ± standard deviation or mean + standard deviation (gene expression data). Triplicates of four donors were used (n = 12). Significance was defined as p ≤ 0.05.

## Results

### hACPCs can be successfully transduced with AD.BMP-2 in three-dimensional culture

**Fig 1:**
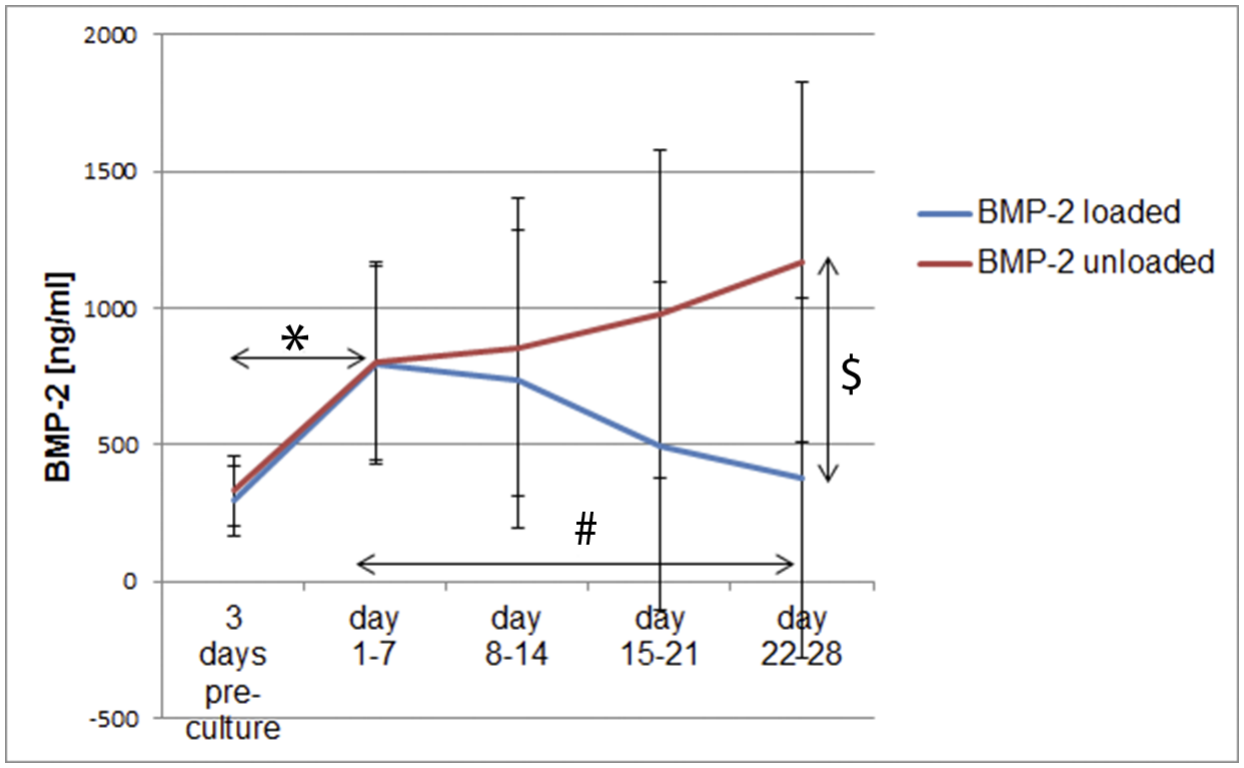
BMP-2 concentration within the cell culture medium for control samples (red) and loaded samples (blue). The BMP-2 concentration was determined using a quantitative ELISA for human BMP-2. The results are displayed as average ± standard deviation (n = 12). *Significant difference from the 3 day pre-culture time-point; # = loaded sample significantly different from day 1–7 time point; $ = significant difference in the unloaded vs. the loaded group, (*p ≤ 0*.*05*).

It already has been demonstrated that hMSCs can be transduced with Ad.BMP-2 in a three-dimensional (3D) environment [29]. Yet, these results are not necessarily transferable to other cell types, such as hACPCs. Therefore, it was investigated if this technique was also applicable for hACPCs. The cells were either left as untransduced controls or transduced with Ad.BMP-2 in 3D with a MOI of 5. The cell culture medium was collected and the BMP-2 concentration was determined by quantitative ELISA. In untransduced samples, BMP-2 medium concentrations were always below the detection level of the assay and, therefore, not included in Fig 1.

After Ad.BMP-2 transduction in 3D, hACPCs started to produce significant amounts of BMP-2 (100 ng/ml or above) (Fig 1). Both groups displayed a statistically significant increase in BMP-2 medium levels between the 3 day pre-culture and the day 1–7 time-point (Fig 1). At later time points, each group displayed opposite trends. In the unloaded group, BMP-2 medium levels stayed relatively consistent. In the loaded group however, the BMP-2 medium levels steadily and significantly decreased between week 1 and week 4. After 4 weeks of culture, BMP-2 medium levels were significantly higher in the unloaded group, if compared to the loaded group (1164.8ng/ml ± 658.6ng/ml vs. 376.7ng/ml ± 206.9ng/ml, *p ≤ 0*.*05*).

### hACPCs endogenously produce TGF-β1 during culture in fibrin-PU composite scaffolds

**Fig 2:**
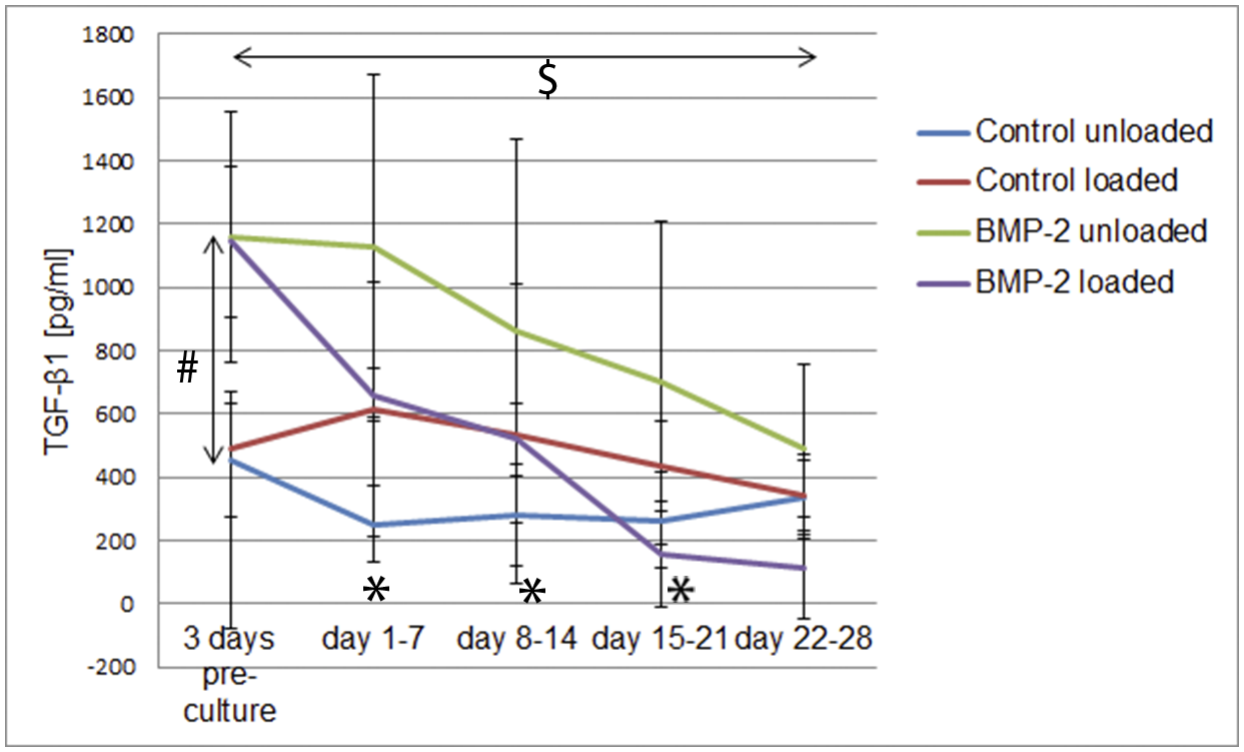
TGF-β1 concentration within the cell culture medium. The TGF-β1 concentration was determined using a quantitative ELISA for human TGF-β1. The results are displayed as average ± standard deviation (n = 12). # = significant difference between the control and the BMP-2 group (both loaded and unloaded); * = significant difference between control unloaded and control loaded; $ = significantly different from the 3 days pre-culture time-point, (*p ≤ 0*.*05*).

It already has been demonstrated that hMSCs are able to endogenously produce TGF-β1, if cultured in medium that lacks exogenous TGF-β1. Further, it has been demonstrated that TGF-β1 production was increased, when these cells were subjected to mechanical stimulation and that this increased endogenous TGF-β1 production was able to induce chondrogenesis [22]. For hACPCs this information is not yet available. Therefore, it was crucial to determine if these cells are able to endogenously produce TGF-β1 and how this production is influenced by mechanical stimulation. The TGF-β1 concentration, within the cell culture medium, was analysed using a quantitative ELISA kit (Fig 2).

In the control unloaded group, the TGF-β1 medium levels stayed relatively stable over time. However, over the first three weeks of culture, significantly increased levels of TGF-β1 were detected in the medium of loaded samples (p ≤ 0.05).

During the 3 day pre-culture period, a statistically significant difference in TGF-β1 medium levels between the control and the Ad.BMP-2 transduced groups was detected (loaded and unloaded). This significant increase was maintained between Ad.BMP-2 unloaded and control unloaded. Both Ad.BMP-2 transduced groups displayed decreasing TGF-β1 medium levels over time, with the decrease being more dramatic in Ad.BMP-2 loaded samples.

### Mechanical stimulation leads to chondrogenic gene expression, which is negatively influenced by BMP-2 overexpression

The effect of mechanical stimulation and transduction with Ad.BMP-2, on the gene expression profile of hACPCs, was investigated. Gene expression analyses were conducted, using the comparative ΔΔC_T_ method, with 18s rRNA as an internal control and normalized to the control unloaded group.

**Fig 3:**
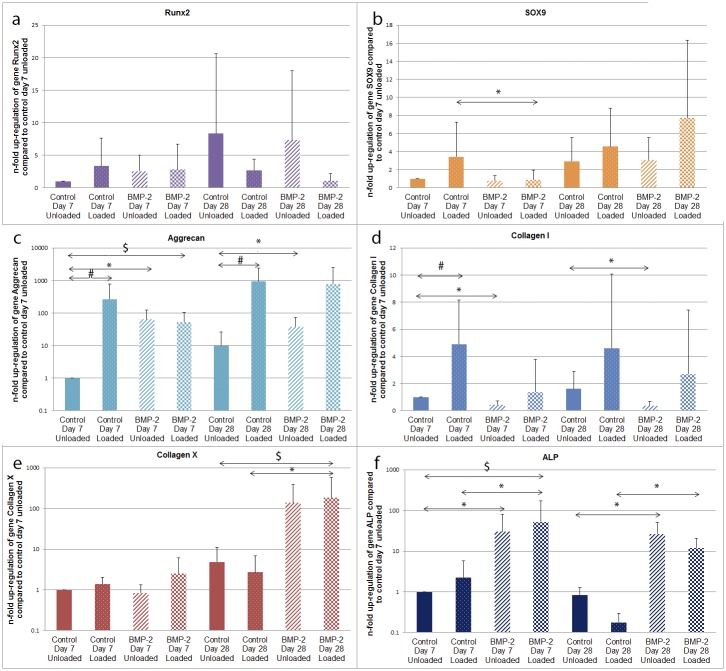
Relative gene expression of hACPCs. Gene expression analyses (a. Runx2, b. SOX9. c. Aggrecan, d. collagen I, e. collagen X, f. ALP) were conducted using the comparative ΔΔC_T_ method and 18s RNA as internal control. * = significant difference between control and BMP-2 (comparing unloaded to unloaded and loaded to loaded); # = significant difference between unloaded and loaded; $ = significant difference between control unloaded and BMP-2 loaded, (*p ≤ 0*.*05*).

For the investigated transcription factors, Runx2 displayed no significant changes in gene expression and little change in SOX9 was detected, although loading conditions did tend towards an increase in SOX9 (Fig 3).

An overview of Col II gene expression can be found in Table 3. Collagen II was mostly undetectable in cells where no stimulus was applied, indicating a lack of spontaneous chondrogenesis under these 3D conditions. Under control loading conditions, Col II expression was consistently detected in all donors at the late time-point (day 28) and was already detected by day 7 in three out of four donors. Co-stimulation with BMP-2 led to a decrease in the frequency of Col II detection under loaded conditions.

**Table 3:** Overview of Col II gene expression within the study. The table displays the groups, in which Col II message was detected within the four runs of the experiment. ND = not detectable.

Col II gene expression detected								
Group	Control day 7 unloaded	Control day 7 loaded	BMP-2 day 7 unloaded	BMP-2 day 7 loaded	Control day 28 unloaded	Control day 28 loaded	BMP-2 day 28 unloaded	BMP-2 day 28 loaded
Run ↓								
**I**	ND	ND	ND	ND	ND	**Yes**	ND	**Yes**
**II**	ND	**Yes**	ND	ND	ND	**Yes**	ND	ND
**III**	**Yes**	**Yes**	**Yes**	**Yes**	**Yes**	**Yes**	**Yes**	**Yes**
**IV**	ND	**Yes**	ND	ND	ND	**Yes**	ND	ND

Mechanical stimulation led to massive and significant up-regulation of the gene aggrecan in the control group at both time-points, when compared to their respective unloaded control counter-part (Fig 3c). Further, transduction with Ad.BMP-2 led to a significant up-regulation of aggrecan message, in the unloaded group on both the early and the late time-point.

Gene expression of the gene collagen type I (Col I) (Fig 3d), was significantly elevated by mechanical stimulation in the control group at day 7. Further, transduction with Ad.BMP-2 led to a small, but significant, down-regulation of Col I message in the unloaded group, at both time-points.

After 7 days, neither mechanical stimulation nor BMP-2 had an effect on Col X expression (Fig 3e). Again, after 28 days, load alone had no effect on Col X message. Contrary to this, adenoviral-mediated over-expression of BMP-2 led to an over 100-fold, significant increase in Col X message in the loaded group on day 28.

Mechanical stimulation did not lead to any significant changes in ALP gene expression (Fig 3f). Even though it led to an 80% decrease in ALP expression, in the load control group on day 28, this trend failed to reach significance *(p = 0*.*097)*. ontrary to this, transduction with Ad.BMP-2 led to a statistically significant increase in ALP gene expression.

**Fig 4:**
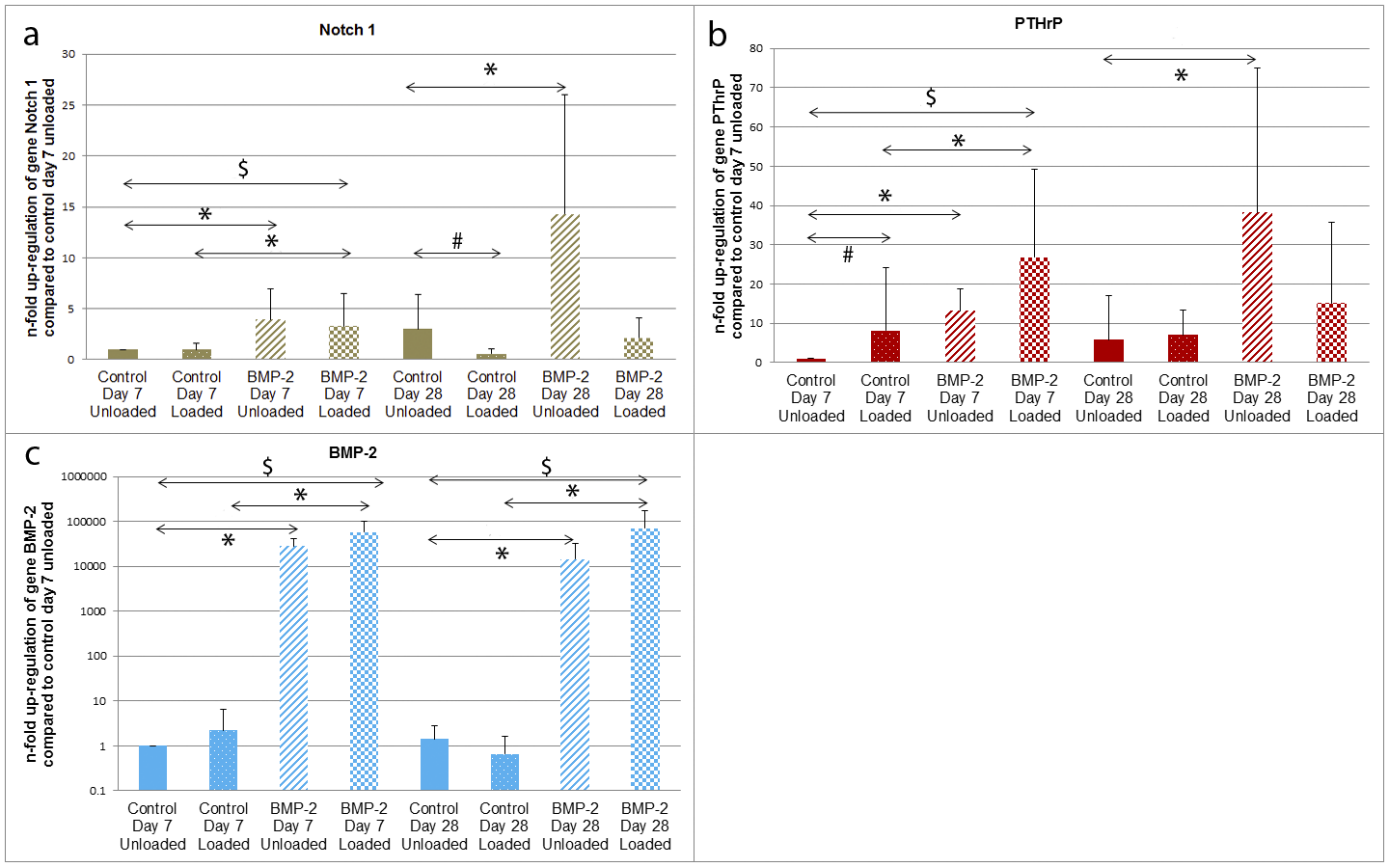
Relative gene expression of hACPCs. Gene expression analyses (a. Notch 1, b. PTHrP, c. BMP-2) were conducted using the comparative ΔΔC_T_ method and 18s RNA as internal control. * = Significant difference between control and BMP-2 (comparing unloaded to unloaded and loaded to loaded); # = significant difference between unloaded and loaded; $ = significant difference between control unloaded and BMP-2 loaded, (*p ≤ 0*.*05*).

Notch 1 gene expression (Fig 4a) was hardly influenced by mechanical stimulation. The only significant, yet very small, change observed was a down-regulation in the control group at day 28. On the other hand, transduction with Ad.BMP-2 led to a significant increase in Notch 1 message in both the unloaded and the loaded group at day 7 and in the unloaded group at day 28. At the early time-point, mechanical stimulation and transduction with Ad.BMP-2 led to a significant increase in PTHrP expression, when compared to the control unloaded group (Fig 4b). Further, PTHrP expression was significantly higher in the BMP-2 loaded group, when compared to the control loaded group. At the late time-point, transduction with Ad.BMP-2 led to a significant up-regulation in PTHrP expression in the unloaded group, when compared to the control group. The gene BMP-2 was un-responsive towards mechanical stimulation (Fig 4c). However, in Ad.BMP-2 transduced cells, BMP-2 message was massively and significantly elevated. This was true for both groups at both time-points.

### Mechanical load increases GAG/DNA, and this increase is reduced by BMP-2

**Fig 5:**
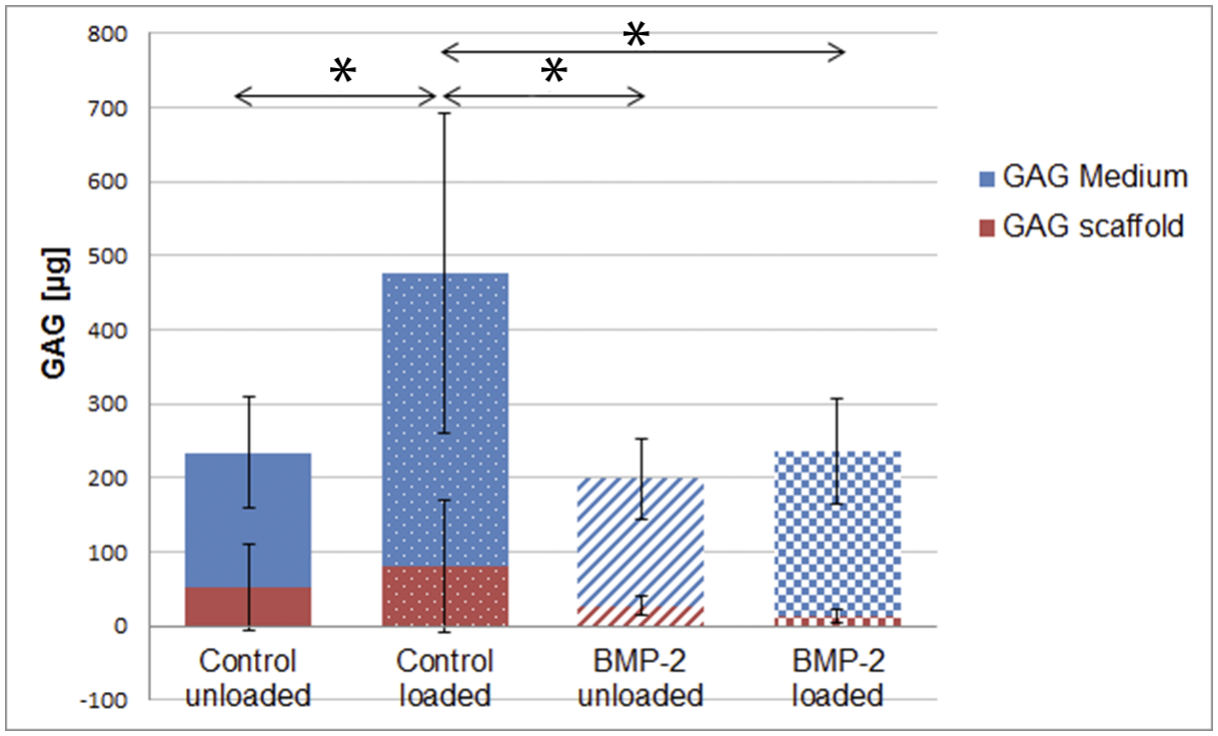
Total GAG (medium + scaffolds) production of hACPCs, seeded into fibrin-PU composite scaffolds, after 4 weeks of culture. The GAG content was quantified using the DMMB dye binding assay with chondroitin-4-sulfate as standard. The results are displayed as average ± standard deviation (n = 12). * = Significantly different from the control loaded group, (*p ≤ 0*.*05*).

The cumulative amount of released GAG was quantified using the DMMB dye binding assay. After 4 weeks of culture, the amount of GAG retained within the scaffolds was measured (Fig 5). Thereby, total GAG production of hACPCs was tracked as a marker of phenotypic change.

The bulk of GAG synthesized was released into the culture medium, which is a known characteristic of the scaffold system used [31]. Mechanical stimulation beneficially influenced GAG production. The control loaded group produced the highest total amount of GAG, which was significantly higher, when compared to the other three groups (*p ≤ 0*.*05)*. Transduction with Ad.BMP-2 significantly reduced GAG production in the loaded group, when compared to the control loaded group. After 4 weeks, a total of 231.4μg ± 107.7μg (control unloaded), 474.2μg ± 240.6μg (control loaded), 196.5μg ± 60.9μg (BMP-2 unloaded) and 233.7μg ± 76.8μg (BMP-2 loaded) of GAG was synthesized.

### GAG/DNA ratio is higher in control vs. Ad.BMP-2 transduced samples and in loaded vs. unloaded samples

**Fig 6:**
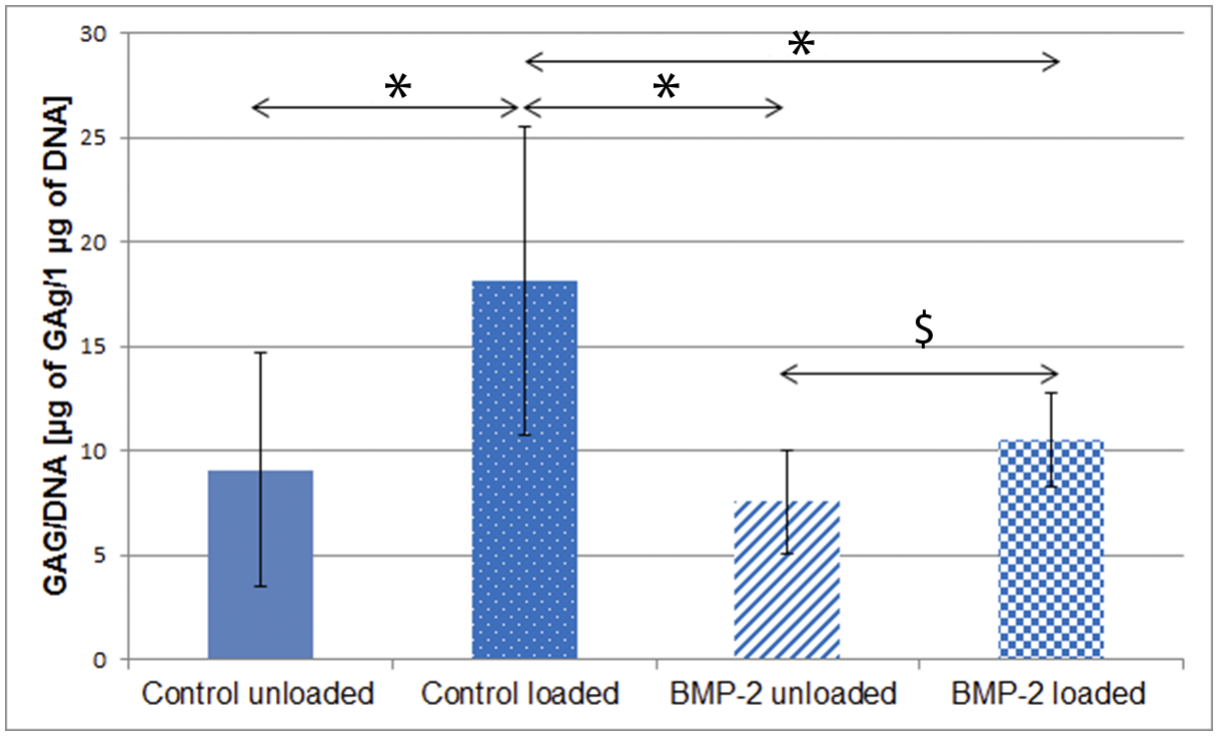
GAG/DNA ratio of hACPCs, after 4 week of culture. The total amount of GAG and the amount of DNA was quantified and GAG production was normalised to DNA content. Results are displayed as averages ± standard deviation (n = 12). * = significantly different from the control loaded group; $ = significantly different from the BMP-2 loaded group (*p ≤ 0*.*05*).

In order to finalize the biochemical analyses, the GAG/DNA ratio was calculated (Fig 6). This procedure normalizes the total amount of synthesized matrix (medium + scaffolds) to the DNA content (indicator for total cell number). Neither mechanical stimulation nor transduction with Ad.BMP-2 led to significant changes in DNA content (data not shown).

In the loaded groups, the GAG/DNA ratio was significantly higher than in the unloaded groups (for both control and BMP-2). However, combining transduction with Ad.BMP-2 and multiaxial load, while still showing an increased GAG/DNA over Ad.BMP2 alone, dramatically reduced the GAG/DNA when compared to load alone. The GAG/DNA ratio was significantly higher in the control loaded group, if compared to the three other groups investigated (*p ≤ 0*.*05)*. The GAG/DNA ratio, in μg /μg, was 9.1 ± 5.6 (control unloaded), 18.16 ± 7.35 (control loaded), 7.56 ± 2.47 (BMP-2 unloaded) and 10.53 ± 2.27 (BMP-2 unloaded).

### Histological evaluation did not detect the presence of glyscosaminoglycan nor collagen II

Safranin O/Fast green and immunohistochemistry for aggrecan and Col II were all negative for all conditions (data not shown). This would suggest that matrix synthesis was limited and the bulk of any protein produced may have been released into the culture medium as demonstrated by the results of GAG quantification.

## Discussion

ACPCs represent a new potential cell source for TE of articular cartilage [19]. Mechanical stimulation influences both the development and the maintenance of articular cartilage. The current study opted to investigate the effect of mechanical stimulation and adenoviral-mediated over-expression of BMP-2, alone or in combination, on the chondrogenesis of monolayer-expanded hACPCs. hACPCs were encapsulated in fibrin and seeded into PU scaffolds. As mechanical stimulation was hypothesised to induce chondrogenesis in hACPCs, a chondro-permissive medium, which lacks exogenous growth factors was used. Mechanical stimulation and transduction with Ad.BMP-2 were applied alone or in combination and the samples were cultured for 7 or 28 days. The strain applied was chosen based on previous studies as it has been shown to result in chondrogenesis of human bone marrow derived MSCs. The choice of strain applied also needs to account for the mechanical properties of the scaffold material, and aims to apply a stimulus, while at the same time leaving the construct undamaged. Further, an untransduced control group was included (cultured under free-swelling conditions). While using an untransduced control, rather than a viral transduced control such as Ad.GFP, could be viewed as a weakness of the BMP-2 part of the study, it has been shown that adenoviral control vectors do not lead to chondrogenesis [30, 31]. For hMSCs, it has been demonstrated that mechanical stimulation leads to an enhanced endogenous production of TGF-β1 and that this production was able to induce chondrogenesis, if exogenous TGF-β1 was omitted from the culture medium [34]. It was hypothesised that hACPCs respond similarly towards mechanical stimulation.

It was confirmed that hACPCs endogenously produce TGF-β1, if cultured in medium lacking exogenous growth factors. Load alone led to a significant increase in TGF-β1 production for the first 3 weeks of culture, demonstrating that hACPCs are responsive to complex multiaxial load. To our knowledge this is the first demonstration of mechano-responsiveness in these cells.

After 3D transduction with Ad.BMP-2, hACPCs were able to produce BMP-2, confirming the feasibility of the novel 3D transduction protocol for hACPCs, seeded into fibrin-PU scaffolds. BMP-2 medium levels were always 100ng/ml or above. This concentration is considered biologically relevant and commonly used when BMP-2 is applied exogenously [35–37]. After one week of similar behaviour, load modified the expression of BMP-2 relative to static conditions. In the loaded group, BMP-2 medium levels steadily and significantly decreased between week 1 and week 4 of culture. After 4 weeks of culture, the BMP-2 concentration within the unloaded group was significantly higher, when compared to the loaded group.

After 3 days of pre-culture, significantly more TGF-β1 was produced in the Ad.BMP-2 transduced samples. This result was true for both the unloaded and the loaded group and indicates that the presence (or production) of BMP-2 enhanced endogenous TGF-β1 production at early time points. After 7 days however, this observation was only repeated in the unloaded group. In the Ad.BMP-2 transduced groups, TGF-β1 medium levels constantly decreased and the TGF-β1 medium concentration in the BMP-2 loaded group, after 4 weeks, was significantly lower, when compared to the 3 days pre-culture time-point. Also, starting from week 2 of culture, no significant differences between the groups were detected.

After 4 weeks of culture, there were no significant differences in DNA content between the different groups. As expected, the GAG quantification revealed that the bulk amount of synthesised GAG was released into the culture medium and not retained within the scaffold. Similar observations were made using fibrin-PU composite scaffolds and hMSCs [25, 34, 38]. Generally, the amount of matrix that can be retained depends on the pericellular matrix, the mechanical environment and the porosity of the scaffolds. The current results suggest that the pericellular matrix of the hACPCs was not mature enough to retain most of the synthesised GAG. This task was further complicated by application of mechanical stimulation and the properties of the PU scaffold (pore size, highly porous scaffold structure). However, by quantifying total GAG production, an accurate assessment of phenotypic change can be obtained and modified phenotype was the main outcome parameter. Regarding total GAG production, the two stimuli had different effects. In the control group, mechanical stimulation led to a significant increase in GAG production. Contrary to this, adenoviral-mediated over-expression of BMP-2 led to a trend towards a decrease in GAG production in both the loaded and the unloaded group. The control loaded group was superior to the remaining three groups and produced significantly more GAG. A similar response to both stimuli was observed when using hMSCs [25].

The normalisation of GAG synthesis to cell number (DNA content) revealed that mechanical stimulation led to an increase in the GAG/DNA ratio. Contrary to the total amount of GAG produced, this increase was significant for both the control and the Ad.BMP-2 transduced group. Furthermore, in the loaded group, adenoviral-mediated over-expression of BMP-2 led to a significant decrease in GAG/DNA ratio. Thus, load led to an increase, whereas transduction with Ad.BMP- 2 led to a decrease in the GAG/DNA ratio. The lack of retention of GAG within the scaffold was evident when investigating the histological sections. While this may be a feature of the scaffold system used, it may also indicate that serum containing medium is a requirement for ACPC matrix synthesis. Previous work with these cells demonstrated matrix production when using medium containing 2% serum in pellet culture [18]. Within this study, serum free medium was employed and matrix synthesis was limited. As maintaining MSC constructs under identical conditions does lead to staining at the histological level, it may suggest that the defined serum free medium used, which has been optimised for MSCs, may be suboptimal for ACPC matrix synthesis, however this would need to be further investigated by performing the study in low serum medium. Alternatively, the central areas of pellet culture maybe more effective at retaining matrix products, thus leading to a more prominent staining pattern. This also shows the necessity not to rely solely on PCR data for a correct interpretation of final construct properties.

Gene expression analyses revealed interesting data that conflicted with the biochemical analysis. In the BMP-2 unloaded group, aggrecan message was significantly up-regulated, when compared to the control unloaded group. Yet, the total GAG production and the GAG/DNA ratio were not different between these groups. Further, on the gene level, there were no differences between the control loaded and the BMP-2 loaded group. Still, the control loaded group had a significantly higher total GAG production and a significantly higher GAG/DNA ratio. This discrepancy between aggrecan gene expression and matrix production has already been observed within our group and by other investigators [31, 39, 40], highlighting the importance of investigating the protein in addition to gene expression changes.

For the gene Col II, mechanical stimulation was beneficial, whereas transduction with Ad.BMP-2 seemed to be detrimental. It has to be clearly emphasised that the only group to consistently express Col II message was the control loaded group on day 28, which highlights the critical influence of multiaxial load on chondrogenic differentiation under serum-free conditions in the absence of exogenous growth factors.

Analysis of the hypertrophic marker gene Col X and the early osteogenic marker gene ALP revealed that mechanical stimulation had no significant effect on these genes. For the gene ALP, transduction with Ad.BMP-2 led to a significant up-regulation of gene expression in both the loaded and the unloaded group. For the gene Col X, exposure to supraphysiological doses of BMP-2, resulted in a massive increase in gene expression, in both groups, after 28 days. For the gene PTHrP, mechanical stimulation and, more predominantly, transduction with Ad.BMP-2, led to an increase in gene expression. This up-regulation can be interpreted as an attempt to counteract a possible hypertrophic differentiation of the cells.

The genes Runx2, Notch 1, SOX9 and Col I were either un-responsive (Runx2) or small changes in gene expression were monitored (Notch 1, SOX9 and Col I), when the stimuli were applied. For the gene SOX9, as it is a transcription factor, it is likely that even these small increases in gene expression led to a significant cellular response.

As expected for BMP-2, it was observed that adenoviral-mediated over-expression of BMP-2 massively and significantly up-regulated BMP-2 gene expression, independent of mechanical stimulation. This massive increase in BMP-2 expression was virally driven and was not related to an endogenous increase in gene expression.

Taken together, the gene expression profile of these genes indicated that both stimuli are, at least to some extent, able to induce a chondrogenic response. Yet, mechanical stimulation resulted in a more stable chondrogenic phenotype (aggrecan high, Col II high, Col X and ALP low). On the contrary, transduction with Ad.BMP-2, independent of the application of load, resulted in a more hypertrophic phenotype (aggrecan, Col X and ALP all high). This would suggest that ACPCs are able of undergoing mechanically induced chondrogenesis to a non-hypertrophic phenotype, but they are able to undergo hypertrophy when exposed to BMP-2 over-expression.

## Conclusion

Summarised, mechanical stimulation was beneficial for *in vitro* chondrogenesis of hACPCs. Biochemical analysis revealed a significant increase in both the total GAG production and the GAG/DNA ratio. Further, the gene expression profile of the control loaded group was very promising, especially concerning Col II data. Compared to the unloaded control group (baseline), the chondrogenic marker genes aggrecan and Col II were massively up-regulated, after 4 weeks of culture. Yet, no sign of an increase in hypertrophic (Col X) or osteogenic (ALP, Runx2) marker gene expression was evident when compared to controls. On the other hand, supra-physiological doses of BMP-2 were not beneficial in modulating the chondrogenic response. Even though this stimulus led to a significant increase in aggrecan gene expression, most of the investigated effects were negative. Biochemical analysis revealed that, the total GAG synthesis and the GAG/DNA ratio were significantly reduced in the Ad.BMP-2 transduced and loaded group, when compared to its corresponding untransduced control. Further, transduction with Ad.BMP-2 led to a significant increase in hypertrophic (Col X) and osteogenic (ALP) marker gene expression, independent of mechanical stimulation. This progression towards hypertrophy is one of the main draw drawbacks of hMSCs, as cell source for cartilage TE [41–43]. More recently, differentiation under hypoxia has been shown to result in a more stable chondrogenic phenotype when using bone marrow MSCs [44]. It would be interesting to compare ACPCs under hypoxic differentiation conditions. Even though the total matrix production of ACPCs was not as high as in hMSCs under these serum free conditions, hACPCs seemed to undergo stable chondrogenic differentiation with no signs of hypertrophic differentiation after differentiation under normoxic conditions. These observations were made under load without over-expression of BMP-2 and consistently led to an initiation of Col II expression. Further, the application of any exogenous growth factor was not necessary, clearly indicating the important role of mechanical stimulation in hACPCs chondrogenesis.
